# Racism and mental health and the role of mental health professionals

**DOI:** 10.1192/j.eurpsy.2021.2216

**Published:** 2021-06-17

**Authors:** M. Schouler-Ocak, D. Bhugra, M. C. Kastrup, G. Dom, A. Heinz, L. Küey, P. Gorwood

**Affiliations:** 1 Psychiatric University Clinic of Charité at St. Hedwig Hospital, Berlin, Germany; 2 Institute of Psychiatry, Psychology and Neuroscience, King’s College London, London, United Kingdom; 3 Specialist in Psychiatryformer Treasurer World Association Social Psychiatryformer Secretary General European Psychiatric Associationformer Member Executive Committee World Psychiatric Association Copenhagen, Denmark; 4 CAPRI, University of Antwerp (UAntwerp), Antwerp, Belgium; 5 Department for Psychiatry and Psychotherapy, CCM, Charité—University Medicine, Berlin, Germany; 6 Istanbul Bilgi University, Istanbul, Turkey; 7 CMME, Hopital Sainte-Anne GHU Paris Psychiatrie et Neurosciences Université de Paris, INSERM U894, Paris, France

**Keywords:** EPA Task Force, mental health, mental health professionals, racism, social construction

## Abstract

The concept of “race” and consequently of racism is not a recent phenomenon, although it had profound effects on the lives of populations over the last several hundred years. Using slaves and indentured labor from racial groups designated to be “the others,” who was seen as inferior and thus did not deserve privileges, and who were often deprived of the right to life and basic needs as well as freedoms. Thus, creation of “the other” on the basis of physical characteristics and dehumanizing them became more prominent. Racism is significantly related to poor health, including mental health. The impact of racism in psychiatric research and clinical practice is not sufficiently investigated. Findings clearly show that the concept of “race” is genetically incorrect. Therefore, the implicit racism that underlies many established “scientific” paradigms need be changed. Furthermore, to overcome the internalized, interpersonal, and institutional racism, the impact of racism on health and on mental health must be an integral part of educational curricula, from undergraduate levels through continuing professional development, clinical work, and research. In awareness of the consequences of racism at all levels (micro, meso, and macro), recommendations for clinicians, policymakers, and researchers are worked out.

## Introduction

Recent racist events in the United States, for example, Black Lives Matter movement [1] in connection with the death of Georg Floyd, have focused the attention of much of the world on the terrible impact of systemic racism on the lives and mental health of communities of color. For mental health professionals, the disastrous psychological consequences of racism and discrimination are obvious. Regarding the link between racism and its impact on mental health, one study showed that, in a representative sample of British citizens, between 26 and 35% of the studied ethnic minorities groups experienced some form of racial discrimination, and ethnic minority people who reported exposure to racial discrimination had lower mental health (SF-12) = −1.93, [−3.31, −0.56] than those who did not [2]. Only a few studies focus on structural racism. Hedden et al. [3] did not find any racial disparities in jail-based treatment, although community-based outcomes significantly differed. Compared with people of color, White people had 1.9 times greater odds of receiving community-based mental health and substance use treatment and 4.5 times greater odds of receiving co-occurring disorder treatment. Pervasive inequities in society favor members of dominant groups, at the expense of racialized minorities, people with diverse gender or sexual orientations, languages or religions. Such exclusion and persecution have a huge impact on mental health, in particular for affective and psychotic disorders as well as substance use disorders. Racism is pervasive and can manifest in several often-overlapping forms (including personal, cultural, structural, and institutional racism), and we cannot be confident about the absence of systemic bias also in psychiatry. Tackling racism and racial discrimination in psychiatry requires an open and frank discussion about how it can be addressed. This happened in the “Position Paper of The Royal College of Psychiatrists” [4] and the “Call to Action on Racism and Social Justice in Mental Health” [5]. The European Psychiatric Association (EPA) sees also a need to become more aware of and sensitive to this issue, and takes necessary action to address its numerous angles. Therefore, the EPA set up a Task Force of experts to prepare this policy statement paper. It provides an overview of different forms of racism and their effects on mental health and well-being, as well as recommendations for clinicians, policymakers, and researchers.

## Definitions of “Race,” Racism, and Ethnocentrism

While human racial groups are not biological categories, “race” as a social construction is very real, both now and throughout human history [6]. Incorrect categorical classifications, such as the term “race,” are regularly and frequently used to discriminate against people and exclude them socially. The original colonial concept was turned into a (pseudo) genetic one to explain the differences. The overwhelming portion of the literature on intelligence, “race,” and genetics is based on folk taxonomies rather than on scientific analyses [6].

Ethnocentrism is the overvaluing of one’s own culture in relation to other cultures, thereby leading to biased judgments. Racism is defined by falsely explaining psychocultural traits as being grounded in categorical biological differences, although there is no valid biological “race” concept, thus proclaiming an inherent superiority of a perceived “race” and its right to domination over others [7,8].

Racism can occur at structural, institutional, interpersonal, and internalized level. It can refer to any number of minority groups, for example, anti-Semitism, homophobia, Islamophobia, and xenophobia [7,9–11]. The construct of “race” is socially important because it underlies social oppression and exclusion [12], which can have strong negative effects on mental health and well-being [9,10,13]. In this line, racial discrimination and exclusion play a causal or mediating role in these mental health problems. It is still the subject of scientific debate [14], and has a central role as a social stressor in the affected groups, which may increase the risk of developing psychotic symptoms [15] and may exacerbate other forms of suffering at micro-, meso-, and macro-levels [16,17]. Micro-level racialization is inextricably framed by the influence of familial socialization and shared cultural values, which are manifest in individuals positioned within various ethnic, classed, and gendered groups. These are themselves shifting rather than static, shaping and shaped by interactions with other identity groups, and influenced significantly by local environmental conditions [17]. Micro-level refers to interpersonal and internalized racism (individual/clinical). Meso-level (professional), institutional racialization recognizes cumulative disadvantage experienced across interrelated welfare experiences (housing, education, employment, etc.), produced through institutions’ routine operations, regardless of the intentionality of individual actors [17]. Macro-level racism includes social structures and ideologies of a society or institution [9,17,18].

Racisms are fundamental causes of observed “race”/ethnic inequalities in risk of severe mental illness and in outcomes relating to severe mental illness. In order to account for these inequalities, it is important to examine the ways in which structural, institutional, and interpersonal racisms operate and mutually constitute one another. Thus, “race” and “races” are social constructions that have no biological counterpart but represent human-made categories of power and dominance.

## The Role of Genetics in the Concept of “Race” and Racism

The concept of “race” has historically been used as a taxonomic categorization based on common hereditary traits (such as skin color) to elucidate the relationship between our ancestry and our genes. This question does not relate only to philosophical, ethical, or political aspects, but also to health ones, as clinicians taking care of patients with mental disorders need to take into account the connections between “race,” ethnicity, genetics, and health [19]. Proponents of maintaining “race” identifiers argue that even if the genetic component of health disparities is small, self-identified “race” or ethnicity is a useful proxy for other correlated nongenetic variables, and to lose the opportunity to explore these would be detrimental to the public; for example, the polygenic risk scores of schizophrenia in two samples with East Asian or European ancestries had reduced scores when transferred across ancestries [20]. Self-identified “race” raises an important issue: racial categories are often not self-identified but imposed by others; they vary with context (e.g., depending on previous colonial rule, a person from the Carribeans may be classified as Hispanic or Black) and, importantly, unlike some other facets of identity, they generally cannot be chosen, picked up, and changed as one’s affiliations change [21].

Human “races” have no taxonomic meaning that warrants granting them an objective biological existence. Indeed, genetic patterns from all four modes of human inheritance (mtDNA, Y-chromosome, X-linked, and autosome), along with protein markers, showed that continental clustering represents no natural classification of humans [22]. Epidemiological research and pharmacogenomic studies indicate that biomedical and statistical values of “race” are generally vanishing when relevant covariates are controlled for geographical and/or social variables. Interestingly, racism per se might directly account for specific morbid traits that are supposed to be associated with race. An analysis of very low birth weight in African American infants was, for example, attributed to the level of maternal lifetime exposure to interpersonal racism [23,24]. Indeed, the genomic and statistical evidence currently available shows that phylogenetic and genetic similarity-based concepts of “race” fail to be applicable to humans even under minimal rational theoretical principles currently accepted in population genetics/genomics [22]. For instance, “race” accounts for 14.2% of the variance in warfarin dosing when not considering other factors. Yet, when pharmacogenomic and relevant biomarkers are taken into account, the statistical value of “race” is markedly attenuated to 0.3% [25].

But how genetics correlate with “races?” It was initially proposed that the genetics of “race” could be mainly explained by geographical aspects, as inferences on population genetics often rely on models that do not take into account the geographical distribution [26]. This would assume that in each geographical region or continent, common ancestry of the local population shapes regional or at least continental genetic variations, resulting in more or less sharp-cut gene “clusters.” However, the hypothesis that attributes the clustering of human populations to “frictional” effects of landform barriers at continental boundaries was tested and considered as empirically incoherent [22]. Instead, human populations have a common ancestry in accordance with the “out of Africa” hypothesis, with highest genetic variability in the regions of origin (Africa) and inclines and declines due to multiple migrations (including Neanderthal and Denisova Humans) and diverse population interactions [7]. Therefore, to describe human variability, Maglo et al. [22] suggest using the term “clines” [27] instead of “clusters.” The link between genetics and “races” was then analyzed the other way round, not bottom-up (i.e., do races have specific genetic settings?), but top-down (i.e., are genes able to distinguish between races?), using statistical cluster-based methods (seeking to group individuals together who are genetically similar). In this context, multilocus genetic markers, about 93% of the total human genetic variation was found at the *individual* level, while 4.3% was apportioned to *continental* regions [28].

The biological concept of “race” is based on false assumptions regarding categorical differences between populations and has to be rejected. Therefore, the implicit racism that underlies many established “scientific” paradigms should be changed. As the French writer Albert Camus said, “*To name things wrongly is to add misfortune to the world.*”

## Racism and Mental Health

A meta-analysis and systematic review underlined that racism is significantly related to poor health, with the relationship being particularly strong for mental health and less robust for physical health [9]. Several studies have highlighted the negative impact of racial discrimination on mental health, particularly in relation to the development of affective, psychotic, and substance use disorders [7,13]. Depression was the most commonly reported outcome, which had the same magnitude of association as the broader category of negative mental health [9]. A more detailed examination of health outcomes indicates a twofold variation in the strength of association between racism and poor mental health (for suicidal ideation, planning, and attempts, for post-traumatic stress and formal post-traumatic stress disorder). The strong association between racism and poor mental health raises questions about the underlying mechanisms by which racism affects health. Being victim of racism can cause brain changes on the dysregulation of cognitive–affective regions, such as the prefrontal cortex, anterior cingulate cortex, amygdala, and thalamus, which share similarities with pathways leading to anxiety, depression, and psychosis [29]. These regions became more active in response to social rejection, and their activity was correlated with self-reported levels of distress. In several studies moderation analyses for participant subgroups showed that age, sex, current educational level, and birthplace do not significantly moderate the association between racism and any of the health outcomes examined. However, the associations of racism with depression, negative mental health, and physical health were significantly different across US ethnic groups [9]. For physical health, US Africans and US Latins were the only groups for which sufficient numbers of associations were reported to allow for moderation analyses. Obviously, chronic exposure to racism may be implicated in the hypothalamic–pituitary–adrenal axis dysregulation that, in turn, can damage bodily systems and lead to physical negative outcomes, such as cardiovascular diseases and obesity [9].

Discriminatory experiences may influence health through the stress responses they engender [30]. Perceived and experienced discrimination produces significantly heightened stress responses and is related to participation in unhealthy behaviors and nonparticipation in healthy behaviors [13,30]. Thus, stigma and social exclusion commonly affect a person’s recovery process as well as their opportunities for societal participation. Improving working conditions, promoting organizational strategies that support coping behaviors, and challenging discrimination will improve mental health [31]. Stigmatization of mental health problems and discrimination of migrants, refugees, asylum seekers, ethnic minorities, and people of color are strong influencing factors of mental health consequences. Additionally, perceived and experienced discrimination is associated with underutilization of medical care services, delays in accessing mental and general medical healthcare, as well as nonadherence to treatment. Furthermore, patient-reported discrimination in the health care setting is associated with worse health, lower satisfaction with healthcare and lower utilization of health services in all racial/ethnic groups in a nationally representative sample of old adults with chronic diseases [32]. Delays in prescriptions and medical tests can lead to seek for alternative health care [33]. Finally, increased diversity among students and trainees may have influenced the culture of health care. They are markers of increased consciousness regarding health equity and justice [34]. The results and meanings of studies on racism depend of the place where the studies have been performed, with the consequence that antiracists approaches will probably benefit of an adaptation process, taking into account the local history, demography, migration patterns, and policy.

## Institutional Racism

Institutional racism is defined as the meso- and macro-level systems, institutions, ideologies, and processes that interact with one another to generate and reinforce inequities among racial/ethnic groups [55]. Institutional racism refers to overt and covert policies, practices, and laws that reinforce racial inequality, white superiority and subordination of certain racial groups in relation to access to resources, opportunities, and power [48]. Institutions follow these unwritten rules and through a number of parameters start to discriminate in employment, investigations, and treatment. An important example of this is the racial residential segregation, which is a fundamental cause of racial disparities in health. For different migrant groups, living in an area with a few people from the same ethnic background is associated with increased incidence of psychosis (the so-called ethnic density effect). Although the evidence of the ethnic density effect on psychosis incidence for second-generation migrants is strong, this is either weak or absent for the first generation [49]. Furthermore, evidence suggests that segregation is a primary cause of racial differences in socioeconomic status (SES) by determining and limiting access to education and employment opportunities. SES remains a fundamental cause of racial differences and variations in health [48]. Thus, workers often live in racially segregated neighborhoods. In these communities, structural factors such as overcrowded housing increase exposure and transmission of infectious diseases, which is compounded by limited access to quality health care through discrimination within the health care system [50]. Furthermore, institutional racism has a high impact on health care disparities and on the gap between the need for and the utilization of mental health services by disadvantaged and vulnerable groups. Discrimination, stigma and shame regarding mental illness, poor knowledge about healthcare systems, and poor language proficiency can lead to poor mental health outcomes in disadvantaged and vulnerable groups [56]. The culture of an institution and unconscious bias of people forming this institution constitute racist attitudes and behaviors, which can be dangerous, irrespective of knowledge. Within an institution, this bias is about gaining and retaining power, irrespective of the qualifications of minority group individuals [8].

Our understanding of how racism and racial discrimination function at an institutional level (i.e., in our structures, institutions, policies, and practices) is related to individual and population health, health outcomes of a group of individuals, including distribution within the subgroup [57]. Institutions are organizations made up of individuals and traditions [8]. The culture of an institution and the unconscious bias of the people who make up that institution make up racist attitudes and behaviors. Institutional racism is found in processes, attitudes, and behaviors that lead to discrimination through unintentional prejudice, ignorance, thoughtlessness, unconscious bias, and racial stereotyping that disadvantage people from ethnic minorities. It refers to the whole institution, including people, to create and reinforce inequalities between ethnic groups (see Table 1 for a summary of the different types of racism and the manifestation of these dimensions). It is about gaining and retaining power, regardless of the qualifications of people from minority groups.

**Table 1.** Summarizing of different types of racism and the definition of these dimensions.

Level	Type of racism	Definition	References	Examples	
Micro: microagentic explanations	Internalized racism	Self-stereotyping: incorporation of racist attitudes, stereotypes, prejudice or discrimination, beliefs or ideologies into one’s worldview	[35–44]	Some evidence indicates that in addition to adversely affecting academic performance, the activation of the stigma of inferiority also leads to increases in blood pressure [35]
		Tends to emphasize individual behaviors and downplay structural constraints		Similarly, Taylor et al have found a positive association between internalized racism and alcohol consumption and psychological distress among African Americans [36,37]
Micro: microagentic explanations	Interpersonal racism	Defined as racially motivated interactions between individuals	[8,16,45–47]	Interpersonal racism refers to the persistence of racial prejudice that negatively affects the doctor–patient relationship [47]
		Term which covers the forms of racism, which most people commonly understand as racism because they are the most visible forms		Patients with migration and refugee backgrounds and ethnic minorities may experience stress and feelings of being trapped or of powerlessness and helplessness, which aggravates the health situation [8]
		Tends to emphasize individual behaviors and downplay structural constraints		
		It covers all interactions or behavior between individuals that are racist or have racist content		
		Covers a range of types of racist incidents, from “microaggressions” to racist name-calling and racial bullying and harassment, to discrimination and racist hate crimes		
Meso–Macro: meso—macrostructural explanations	Institutionalized racism	Social and cultural forces, institutions, ideologies and processes that interact to create and reinforce inequalities between ethnic groups	[3,6,8,9,13,31,47–51]	One overview study examined medical articles on pain and found patients of color were more likely to have their pain taken too lightly and less likely to have it medically recorded accurately than white patients [51]
		Culture of an institution and the unconscious bias of the people who make up that institution constitute racist attitudes and behaviors that are more dangerous		
		It is about gaining and retaining power, regardless of the qualifications of people from minority groups		
		Forms of racism expressed in the *practice* of social and political institutions; to the way, institutions discriminate against certain groups, whether intentionally or not, and to their failure to have in place policies that prevent discrimination or discriminatory behavior		
		It can be found in processes, attitudes, and behaviors, which lead to discrimination		
		Through unintentional prejudice, ignorance, thoughtlessness, unconscious bias and racist stereotyping, which disadvantages ethnic minority people		
		Relates to the entire institution, including people		
		Create the conditions that make forms of individual racism seem normal and acceptable, making discrimination and violence more likely		
Macro: macrostructural explanations	Structural racism	Focus on the structural conditions that influence individual behavioral dispositions set of ways in which societies promote racist discrimination through mutually reinforcing systems of housing, education, employment, income, benefits, credit, media, health care, and criminal justice	[3,4,6,9,10,14,15,18,28,31,47,51–54]	Evidence that racism appears to have twice the impact on mental health (for suicide ideation, planning and attempts, and post-traumatic stress disorder). For example, specific negative mental health effects of perceived and experienced discrimination and exclusion demonstrated for people from migrant and refugee backgrounds and ethnic minorities, particularly in relation to the development of affective and psychotic disorders and substance use disorders [3,4,6,10,14,15,28]
		Involves interconnected institutions whose linkages are historically rooted and culturally reinforced		
		Reinforcement of discriminatory beliefs, values, and distribution of resources through these patterns and practices		

## Structural Racism

Racism is a multidimensional concept [52] with a negative attribution made by people of one social group toward people of a different group. It delineates systemic racism as a set of policies and structures that spawn toxic environments and identifies behavioral and physiological pathways that mediate the social and spatial concentration of disease [53].

Structural racism is defined as the macro-level systems, social forces, institutions, ideologies, and processes that interact with one another to generate and reinforce inequalities among racial and ethnic groups [55]. It refers to the ways in which societies foster racial discrimination through mutually reinforcing systems of housing, education, employment, earnings, benefits, credit, media, health care, imprisonment, and juvenile custody [54]. Furthermore, these patterns and practices reinforce discriminatory beliefs, values, and distribution of resources [10]. Structural racism, the system-level factors related to interpersonal racism, increases mortality and reduces overall health and well-being [18]. It is a threat to the physical, emotional, and social well-being of every individual in a society that allocates privilege based on “race” [58]. The term “structural racism” emphasizes the most influential socioecologic levels at which racism may affect racial and ethnic health inequalities. It does not require the actions or intent of individuals [59]. It is present both in institutions, social structures and tacit modes of perception and social interaction could be brought out more clearly, this is important in clarifying that racism can persist even when individuals are not overtly or intentionally racist [57]. Even if interpersonal discrimination is completely eliminated, racial inequalities would likely remain unchanged due to the persistence of structural racism, to which many other forms of racism belong, including the prison industrial complex, historical trauma, emotional rules, and media portrayals [60]. This means that research on structural racism should not only focus on independent effects but should also address interactions among multiple forms of racism [18].

## Internalized and Interpersonal Racism

Internalized racism is defined as the incorporation of racist attitudes, stereotypes, prejudices or discrimination, beliefs or ideologies, into one’s worldview. Members of stigmatized racial populations respond to the pervasive negative racial stereotypes by accepting these to be true. This endorsement of the dominant society’s beliefs about their inferiority is seen as internalized racism or self-stereotyping. Inevitably, this can lead to lower psychological well-being and self-esteem and has been associated with higher levels of alcohol consumption, depressive symptoms, and obesity [42]. Furthermore, it can lead to self-injurious incorporation of racist stereotypes about the self, adverse effects on those who experience it. For example, high internalized racism scores have been linked to poor health outcomes among Caribbean black women, higher propensity for violence among African American young males [43,44].

Interpersonal racism is defined as the interactions between individuals at individual levels. Discrimination at the individual level in the form of everyday microaggressions can occur up to exceptionally stressful events with potentially traumatizing effects and can have an individual impact that promotes disease [45]. Given the diverse and often very subtle forms that interpersonal racism takes, it is difficult to quantify the level of risk faced by ethnic minority people [46]. Mental health professionals may carry their own prejudices and unconscious bias toward other “races” and cultures. Furthermore, on occasion they may illustrate what has been described as missionary racism, that is, that the professional knows what is good for the minority individuals who cannot think for themselves. Missionary racism occurs when a person assumes that minorities are unable to think for themselves and thinks on behalf of minority communities [8]. Socioeconomic and educational status contribute to a degree of mitigation whereas stress, feeling trapped or powerless may exacerbate effects of racism on disadvantaged and vulnerable groups.

At an individual level, mental health professionals may also stereotype other backgrounds and unwittingly reject individuals of these groups. The process of marginalization can reduce the identity of the individual to that of a migrant or foreigner and thus, may contribute to a sense of feeling devalued. Migrants, refugees, asylum seekers, ethnic minorities, or people of color may be more visible and therefore vulnerable to being attacked and stereotyped [8,61].

## How to Overcome Institutional, Internalized, and Interpersonal Racism in Mental Health Care

Social determinants of health are important factors to bear in mind. As the Equality and Human Rights Commission [62] highlighted, an individual from Black, Asian, or minority background is more likely to experience poverty, to have poorer educational outcomes, to be unemployed and to come in contact with the criminal justice system. These variables may show cumulative exposure to racial discrimination with incremental negative long-term effects on the mental health of ethnic minority people [13,29]. Even if institutionalized racism and its effects are increasingly reported in the news, television, and social media, its presence in research is often ignored. Researchers need to gather more comprehensive data on the effects of institutionalized racism by analyzing residential segregation, mass incarceration, housing discrimination, concentrated poverty, food insecurity, and other relevant factors. To understand how institutionalized racism affects health and well-being, innovative methods and measures are needed to capture data on the exposure to and health effects of institutionalized racism [63]. These should ultimately challenge the implicit biases that perpetuate institutionalized racism. Physiological and public health consequences of interpersonal racism are well studied, but there is little investigation in the mental health on how institutionalized racism influences outcomes [63]. Therefore, to find effective solutions, the first step is to measure institutionalized racism in a systematic way. Thus, clear focused research on institutionalized racism and its impact on mental health is urgently needed to overcome its substantial, longstanding effects on health and well-being.

## Recommendations

Mental health professionals are enmeshed in society and institutions and are thus just as likely to unknowingly harbor unconscious biases, interpersonal and internalized racism as anyone else. They need to advocate against racism as they bear a great responsibility to provide good healthcare and be good role models. Recognizing and naming racism, in the work, writing, research, and in interactions of mental health professionals with patients and colleagues will advance understanding of the distinction between “race” and racism and will allow for efforts to combat racism [63].

On the bases, we make the following recommendations, keeping in mind that racial discrimination of any group designated to be “the other” in a society increases the risk of discrimination in general.

### For clinicians (all mental health professionals)

1. Racism as a serious risk and stress factor for mental health and well-being has to be addressed in all disciplines. It is crucial that the role and impact of racism on the health and well-being become an integral part of training curricula across different disciplines from undergraduate (including medical students) to postgraduate, and that it becomes a key aspect of continuing vocational training. These aspects should be addressed in a cross-methodological way through, for example, case studies, role plays, patient presentations, supervision, and so forth.
2. Recognizing and naming racism in the work and in interactions of mental health professionals with patients and colleagues will advance understanding of the distinction between “race” and racism. Speak-up guardians should be supported.
3. The identification of racism in interprofessional everyday practice enables efforts to combat racism to raise awareness of racial discrimination, exclusion, and the risk of unequal treatment.
4. Anti-racism training for all occupational groups should be offered as a qualification measure for implementing in quality management (e.g., embedding in cultural competence training).
5. Treatment guidelines in the domain of mental health and well-being should take cultural specificities into account.
6. Consideration must be given to institutions being awarded certificates of excellence or charter marks if they achieve certain standards in equity and equality for staff and patients.
7. Working in interprofessional/multidisciplinary clinical teams in order to generate richer suggestions for overcoming the impact of racism in the case of specific patients and to expand clinicians’ role as patient advocates, for example, in order to address specific social determinants of mental health involving employment, education, housing, and so forth. This could include the value of incorporating individuals with lived experience (“peer providers”) into these expanded clinical teams.

### For policymakers

1. When people are encouraged to go on anti-racism training their response is that they are not racist and therefore, do not require any anti-racist training. Under these circumstances, broader training of all health professionals in cultural competence becomes highly relevant. Cultural competence is a concept for overcoming sociocultural differences and other systemic challenges to reduce disparities with regard to mental health care provision.
2. Training in cultural competence needs to focus on anti-racism practices very explicitly and to conduct a rigorous examination of current policies and practices to identify areas for improvement.
3. Consideration to members of minority communities should be given when filling positions in mental health institutions to demonstrate equal treatment in recruitment and retention.
4. Mental health institutions should appoint a leader for “race”-related issues to monitor the processes from data collection to appointments and retention of staff.
5. Institutions should have and demonstrate clearly anti-racist policies accompanied by actions. This should be visible on their website, networks, and collaborations.
6. Institutions must include elimination of racism as part of their core values, as depicted in their mission statements and policies. Regulatory bodies should identify standards of care for disparate groups of patients and award recognition to those who achieve these standards.
7. The topic of racism and mental health should be included in regular teaching through conferences, workshops, and educational programs of the EPA, National Psychiatric Associations (NPAs), and other institutions to increase awareness.

### For researchers

1. Researchers should gather more comprehensive data on the effects of institutional racism by analyzing residential segregation, mass incarceration, housing discrimination, concentrated poverty, food insecurity, migration, and other relevant factors.
2. Minority patients should not be excluded from research studies.
3. Specific focused research dealing with racism in mental health system must be encouraged and appropriately funded.
4. Research on structural racism should not only focus on independent effects but should also address interactions among multiple forms of racism.
5. Research on institutionalized racism and its impact on mental health are urgently needed in order to overcome its substantial, longstanding effects on health and well-being.
6. By explicitly identifying institutionalized racism in the peer-reviewed literature, researchers can highlight how injustice and discrimination have been codified, reinforced, and propagated in the mental health system.
7. Research on different forms of racism (interpersonal, institutional, and structural, as well as internalized: on micro-, meso-, and macro-levels) and their interactions is needed.
8. Research on the impact of racism, including not only health services research and clinical trials but also biological and sociological mechanisms of illness production and course modification.
9. Recognizing and naming racism in research and writing will advance understanding of the distinction between “race” and racism, and allow for efforts to combat racism when it is recognized and clearly defined. Publications could be analyzed by independent reviewers for racist biases.
10. Racial discriminatory research projects should be excluded from funding. Proposals could be analyzed by independent reviewers for racist biases.
11. Researchers should emphasize the need for research on intervention development to address the impact of racism with individual patients and with health care systems as a whole.
12. Researchers should emphasize the need to expand the racial/ethnic composition of journal boards, reviewer panels, and top editors.

## Conclusions

The concept of “race” and consequently of racism is not a recent phenomenon and human racial groups are not biological categories, while “race” is a social construction. Racism is significantly related to poor health, with the relationship being particularly strong for mental health and less robust for physical health. It is a serious structural, institutionalized, and interpersonal risk and stress factor for mental health and well-being, which has to be addressed in all disciplines. The Task Force of the EPA developed recommendations for clinicians, policymakers, and researchers, keeping in mind that racial discrimination of any group designated to be “the other” in a society increases the risk of discrimination in general.
